# Zein/Polysaccharide Nanoscale Electrostatic Complexes: Preparation, Drug Encapsulation and Antibacterial Properties

**DOI:** 10.3390/nano14020197

**Published:** 2024-01-15

**Authors:** Elena-Daniela Lotos, Marcela Mihai, Ana-Lavinia Vasiliu, Irina Rosca, Alice Mija, Bogdan C. Simionescu, Stergios Pispas

**Affiliations:** 1 Petru Poni Institute of Macromolecular Chemistry, 41A Grigore Ghica Voda Alley, 700487 Iasi, Romania; daniela.lotos@icmpp.ro (E.-D.L.); vasiliu.lavinia@icmpp.ro (A.-L.V.); rosca.irina@icmpp.ro (I.R.); bcsimion@icmpp.ro (B.C.S.); 2 Institut de Chimie de Nice, Université Côte d’Azur, UMR CNRS 7272, 28 Av. Valrose, 06108 Nice, France; alice.mija@univ-cotedazur.fr; 3 Theoretical and Physical Chemistry Institute, National Hellenic Research Foundation, 48 Vassileos Constantinou Ave., 11635 Athens, Greece

**Keywords:** nanoparticles, zein, polysaccharides, antibacterial, ciprofloxacin

## Abstract

Characterization of zein aqueous solutions, as a function of the ethanol content and pH, was performed, giving information on the zein aggregation state for the construction of complexes. The aggregation state and surface charge of zein was found to depend on the mixed solvent composition and pH. Nonstoichiometric complex nanoparticles (NPECs) were prepared by electrostatically self-assembling zein, as the polycation, and sodium alginate or chondroitin sulfate, as the polyanions, at a pH of 4. A wide range of parameters were investigated: the alcohol–water content in the zein solutions, the charge molar ratios, the solution addition order and the addition rate. The resulting nanoparticles were characterized by dynamic and electrophoretic light scattering, circular dichroism and scanning electron microscopy. The smallest size for the NPECs (100 nm) was obtained when the polysaccharides acted as the titrate with an addition rate of 0.03 mL·min^−1^. The NPECs with the best characteristics were selected for loading with ciprofloxacin and then deposited on a cellulosic material in order to evaluate their antibacterial activity. Substantial drug encapsulation with desired drug release profiles were found together with notable antibacterial efficiency, showing the tunability of the properties for both the zein and its complexes with polysaccharides, together with their application potential in the biomedical field.

## 1. Introduction

Plant proteins, like zein [1,2], gliadin [3], glutenin [4], soy proteins [5] and others, are used for various applications in the field of nanotechnology [6]. Zein, a hydrophobic protein from corn, is a suitable material for creating delivery systems, such as micro- and nanoparticles [7,8]. The hydrophobic nature of zein is ascribed to its high content of nonpolar amino acids, such as leucine, alanine and proline [9]. However, zein particles used as the sole material have limited applications because of their tendency for aggregation (poor colloidal stability) and insolubility in water at a physiological pH [10]. To overcome these inconveniences, polysaccharides (PZs) can be incorporated into the zein particles [7] to obtain composite particles and materials with superior characteristics.

Regardless of their provenance, PZs contain repeating-saccharide-based monomeric units. One of the many advantages of PZs is the availability of a wide range of functional groups, allowing for the formation of complexes through electrostatic interactions with other biological molecules, such as proteins [11]. The complexes are usually formed between an anionic PZ and a protein, at a pH value below the isoelectric point of the protein, through electrostatic interactions [12]. Sodium alginate (ALG) is a natural anionic PZ extracted from brown algae, which contains α-L-guluronic and β-D-mannuronic units, with broad applications in the food industry [13,14], pharmaceutical encapsulations [15], tissue engineering [16], wound healing [17] or the textile industry [18] due to its high biocompatibility and biodegradability, low cost and ease of combination with other components. Chondroitin sulfate (CSA) is a natural anionic polymer consisting of alternating disaccharide units of glucuronic acid and galactosamine. Since it is biocompatible, biodegradable, nontoxic and has anti-inflammatory properties, CSA is widely used in medical applications, such as drug or gene delivery [19,20,21] or tissue engineering [22,23].

Polyelectrolyte complexes (PECs) formed by mixing synthetic polyelectrolytes were intensely studied in recent years, considering their facile preparation and responsiveness to environmental stimuli. Nonstoichiometric PECs as colloidal dispersions (NPECs), having an excess of negatively or positively charged polymers, at relatively low concentrations have already found diverse applications in water cleaning (mainly as flocculants [24]), surface property modification [25,26] and medical uses (nanocarriers for active molecules [27]). Our previous studies dealt with the preparation of NPEC nanoparticles as stable colloidal dispersions using strong or weak synthetic or natural (chitosan) polyelectrolytes [28,29]. The changes in the particle size, morphology and storage stability were also investigated, depending on the polyion structure and molar mass, the molar ratio between charges, the addition order and the addition rate [30]. Nevertheless, the NPECs of natural polymers are less studied because of their more complex chemical structure, the variation in their properties according to the source and the production date, as well as their less-flexible macromolecular backbones.

The already-studied methods used to interact zein with polysaccharides are by covalent (with crosslinking agents or involving the Maillard reaction) or noncovalent binding (antisolvent precipitation, pH- or heat-inducted antisolvent precipitation and antisolvent coprecipitation) [7,31]. Herein, zein water/alcohol solutions were used in the formation of hybrid protein–polysaccharide nanoparticles using anionic PZs, chondroitin sulfate (CSA) and sodium alginate (ALG) following the electrostatic interactions between the zein and PZs. To find the proper condition for the NPEC preparation, the dispersion sizes and electrokinetic properties of the zein in alcohol–water mixtures with different alcohol concentrations and at different pHs were first investigated. Nanoparticles of NPECs of different molar ratios between the complementary charged macromolecules (from 0.4 to 1.8) were synthesized by both complementary addition methods (zein to PZ or the reverse order of addition) and at different addition rates (0.03 or 0.08 mL·min^−1^). The obtained zein/PZ nanoparticles were characterized by scanning electron microscopy (SEM), dynamic light scattering (DLS) and circular dichroism (CD). The electrokinetic study and nanoparticle size measurements highlight the role of the constituent polymers (biopolymer structure) and the preparation conditions (the biopolymer addition order, pH, the ratio between biopolymers, the alcohol concentration in the zein solutions and the addition rate) on the NPEC nanoparticle properties. Also, the NPEC nanoparticle capacity to entrap drugs was tested with different contents of ciprofloxacin (CF). The antibacterial activity of the NPEC vs. NPEC/CF nanoparticles on the nonwoven cellulosic material was also tested.

## 2. Materials and Methods

### 2.1. Materials

Zein, ALG from brown algae (viscosity ~50 cps) and CSA from bovine trachea were purchased from Sigma-Aldrich (St. Louis, MO, USA). CF, as hydrochloride monohydrate, from Thermo Fisher Scientific (St. Louis, MO, USA), and ethanol, hydrochloric acid and sodium hydroxide, from Sigma-Aldrich, were used without further purification.

### 2.2. Solution Characterization

Solubility tests were performed for the zein in aqueous ethanol mixtures with different alcohol concentrations (60–90%) and at different pHs (pH = 2–9). The zein aqueous ethanol mixtures were denoted herein, taking into account the alcohol content, Zxx; for example, Z60 is the zein solution in the mixture of 60% ethanol and 40% water. The PZs, i.e., CSA and ALG, were solubilized in distilled water and at a pH range of 2–9. The characterization of the zein and PZ solutions was implemented to find the proper conditions for the preparation of the protein–PZ NPECs.

### 2.3. Synthesis of the NPEC Nanoparticles

Stock solutions of 1 g·L^−1^ zein in an alcohol–water mixture (60 and 70% ethanol, coded in this study as Z60 and Z70, respectively) and 0.1 g·L^−1^ ALG and CSA in distilled water were first prepared. All the solutions were adjusted to a pH of 4 using 0.1 M HCl, and the ionic concentration (molar mass charges) was determined by polyelectrolyte titration. The NPEC nanoparticles used in this study were obtained through both addition pathways: zein to PZ or PZ to zein. Thus, a specific volume of zein or PZ (the titrate, 10^−5^ M ionic units) was introduced in a backer and was titrated with a specific volume of the PZ or zein (the titrant, 10^−4^ M ionic units), drop-by-drop, with addition rates of 0.03 or 0.08 mL·min^−1^ under magnetic stirring up to the reaching of the specific charge molar ratio between the titrant and titrate (n(+/−) or n(−/+)). The obtained NPEC dispersions were characterized and used after 24 h of storage. The sample codes (summarized in Table 1) denote the NPEC components (Zxx and PZ) in the addition order (titrant/titrate), followed by their charge molar ratio; for instance, (Z60/CSA)0.6 is the sample prepared with the CSA 10^−5^ M titrated with Z60 10^−4^ M up to the reaching of the charge molar ratio of 0.6.

### 2.4. NPEC Nanoparticle Interaction with Ciprofloxacin

The NPEC nanoparticle interaction with drug molecules was tested with CF. The same volumes (10 mL) of freshly prepared complexes, (Z70/CSA)0.8 and (Z70/ALG)0.8, were titrated under magnetic stirring and consuming different volumes (1, 2, 5, 7, 10 and 15 mL) of the 10^−4^ M CF solution. After the titration, the obtained dispersions were stirred for 1 h, and then they were left to rest for another hour before the characterization. To determine the concentration of the CF in each solution, the absorbance was measured at λ = 278 nm with a SPEKOL 1300 spectrophotometer (Analytik Jena GmbH, Jena, Germany). The measurements were performed in duplicate in a quartz cuvette.

### 2.5. NPEC and NPEC/CF Deposition on Nonwoven Cellulosic Material

For the deposition of the NPEC and NPEC/CF, the support material of the nonwoven cellulosic fibers (70% wood pulp and 30% cotton, purchased from Sano Romania SRL, Pantelimon, Romania) was first cut into squared equal pieces of 1 cm^2^ and then immersed for 1 h in the 10^−4^ M Z70 solution. Next, the support samples were removed from the solution, dried on a paper towel and immersed in the NPEC or NPEC/CF solutions for another hour, then dried for 24 h and finally tested for antibacterial activity.

### 2.6. Characterization Methods

The particle size and the polydispersity index (PDI) were evaluated by dynamic light scattering, and the apparent zeta potential (ζ_app_) of the nanoparticles was evaluated by electrophoretic mobility using the Zetasizer Nano ZS (Malvern Instruments Ltd., Malvern, UK) at a 173° angle with a 10 mW He-Ne laser source at λ = 633 nm. The reported results are the average of three independent measurements.

A particle-charge detector (PCD 03, Mütek GmbH, Neckartailfingen, Germany) was used to determine the charge density of the polymers by polyelectrolyte titration. The zein and PZ solutions were titrated using standard solutions of 10^−3^ M poly(sodium ethylenesulfonate) or 10^−3^ M poly(diallyldimethylammonium chloride). The zein and PZ molar concentration in the charged groups in the solutions was calculated from the amount of standard solution needed to reach the zero value of the streaming potential.

The conductometric titrations of the zein alcohol/water solutions were performed with a SevenExcellence pH/Cond meter S470-Std-K (Mettler-Toledo GmbH, Greifensee, Switzerland). The electrolytic conductivity of the reaction mixtures of the zein (20 mg) and NaOH (1 mL, 0.07 M) in excess was continuously monitored as the HCl was added. The equivalence points, where the conductivity undergoes sharp changes, were identified as the average volumes (V1, V2 and V3) of HCl (0.1 M).

Scanning electron micrographs were obtained at an accelerating voltage of 5 kV and at various magnifications using the Verios G4 UC scanning electron microscope (Thermo Scientific, Brno, Czech Republic). Samples of the zein/alginate NPECs were deposited on silica wafers and then coated with 6 nm platinum with a Leica EM ACE200 sputter coater.

The evaluation of the secondary structure of the zein was performed using a Chirascan™ CD Spectrometer (Applied Photophysics Limited, Leatherhead, UK). The spectra were acquired in the wavelength range between 190 and 340 nm at 25 °C. The baseline was determined with the solvent used for each sample. For the acquisition of the spectra, 3 mL of each sample were added into a quartz cuvette. The content of α-helix was determined with the BeStSel web server [32,33].

### 2.7. Antibacterial Activity

The antibacterial activity screening of the samples was determined by a disk diffusion assay [34,35] against 2 different reference strains: Staphylococcus aureus ATCC25923 and Escherichia coli ATCC25922. Both microorganisms were stored at −80 °C in 20% glycerol and were refreshed on trypticase soy agar (TSA) at 37 °C prior to the analysis. Microbial suspensions were prepared with these cultures in sterile solution to obtain a turbidity optically comparable to that of the 0.5 McFarland standards. Volumes of 0.1 mL from each inoculum were spread onto TSA plates, then the sterilized cellulosic materials were added. To evaluate the antibacterial properties, the growth inhibition area was measured under standard conditions after 24 h of incubation at 37 °C. The samples were analyzed with SCAN1200^®^ version 8.6.10.0 (Interscience, Puycapel, France), and the measurements were expressed as the mean ± standard deviation (SD), performed with the XLSTAT Ecology software, version 2019.4.1. All tests were carried out in triplicate to verify the results.

## 3. Results and Discussion

### 3.1. Zein Characterization in Solution as a Function of the Alcohol Content and pH

**Figure 1:**
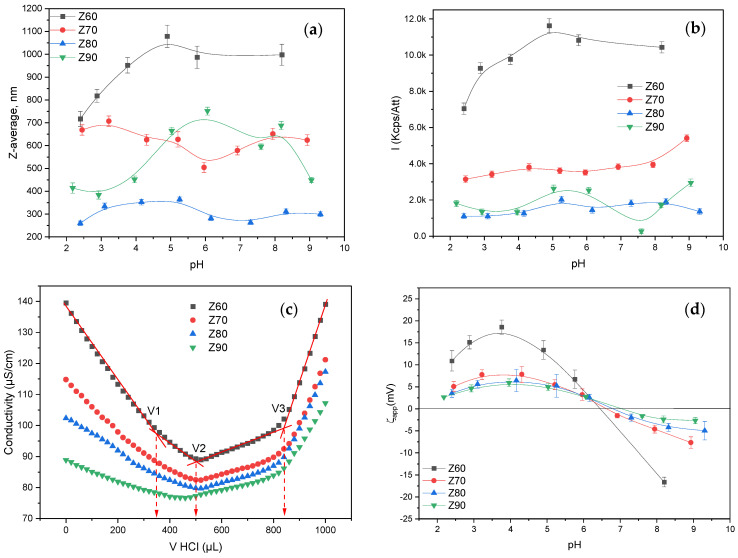
Variation in the (**a**) size (Z-average), (**b**) intensity, (**c**) conductivity titration curves and (**d**) apparent zeta potential (ζ_app_) for the zein solutions with different alcohol concentrations. Error bars were calculated based on three independent measurements.

As is already known, zein is not soluble in water, but is soluble in alcohol/water mixtures [36]. Herein, solutions with various alcohol/water concentrations (with an alcohol content from 60 to 90%) were prepared, and the zein dispersion size (Z-average), scattered light intensity (I) and apparent zeta-potential (ζ_app_) values of each solution were measured at a pH between 2 and 9, along with the conductivity titration curves (Figure 1).

As can be observed in Figure 1, both the alcohol/water ratio and pH influenced the zein characteristics. Thus, for a low alcohol content (Z60 and Z70), the DLS curves for the hydrodynamic diameter presented just one particle population (Figure S1) with a relatively low polydispersity, even if the mean diameter of the resolved peak (Figure S2) and the Z-average (Figure 1a) values were relatively high, in the range of 500–1000 nm. The increase in the alcohol content to 80% and 90%, respectively, resulted in the decrease in the Z-average (Figure 1a), even if two and three populations of particles were found (Figure S1). Nevertheless, the variation in the Z-averages and individual peak mean diameters were less influenced by the pH variation, irrespective of the alcohol/water ratio. At the same time, the scattered light intensity values (Figure 1b) were also less influenced by the pH value, with the alcohol content being the leading parameter for the intensity ranking, and with the highest values being found for less alcohol content, whereas lower and almost-similar values were found for the Z80 and Z90, respectively. The above results indicate that the zein aggregates had a compact structure at a low alcohol content, with large dimensions and high mass, while they became looser upon the increase in the alcohol content, with smaller dimensions and mass.

The acid–base functional groups of the zein are mainly the amino and carboxyl groups present in the different amino acids which form the zein macromolecule chain (Figure S3). The decrease in the conductance can be associated with the changing concentrations of the two most highly conducting ions, the hydronium and hydroxyl ions. Thus, the conductometric back-titration curves of the solutions with the same zein concentrations, but with different ethanol contents (Figure 1c), showed nearly the same inflexion points, which corresponded to three equivalence points (V1, V2 and V3 HCl volumes). Due to the difference in the dielectric constants of the ethanol (29.4) and deionized water (79), the titration curves shifted as a function of the alcohol/water ratio, with the solution conductivity decreasing with the increasing ethanol content. Thus, as shown in Figure 1c, in the case of the Z60 solution, the first equivalent point located to point V1 = ~350 μL (as the intersection point of the linear segments of the curve) was assigned for the neutralization of the excess of NaOH which remained in the solution after the reaction with the acidic groups of the zein. The difference between the initial added volume of the NaOH (1 mL) and the titrated excess (0.500 mL) was attributed to the consumed NaOH (0.500 mL) by the zein amount (20 mg). Therefore, using this volume (0.500 mL), it is possible to evaluate the concentration of the zein acidic groups (1.75 meq/g). Further, the difference, V2–V1 = ~0.150 mL, corresponded to the amount of acid necessary to neutralize the zein amino groups (-NH_2_, -NHR and -NR_2_), with the calculated concentration for the basic groups being 0.75 meq/g. From the third (V3) and second (V2) equivalence points, the volume of acid (0.34 mL) necessary to neutralize the carboxylic sodium salts (-COONa) was calculated, with the calculated concentration being 1.7 meq/g. This value obtained for carboxylic sodium salts (ionized groups) was nearly the same as the calculated value for anionized carboxylic groups, which was 1.75 meq/g. The fourth linear branch of the curves indicated the presence of an excess of a strong electrolyte (HCl). These results confirmed the fact that zein is a polyampholyte with more weak acidic than basic groups, with the molar ratio between the functional groups being 2.33 = 1.75 meq(−)/g: 0.75 meq(+)/g.

For the same alcohol content, the zein changed its ionization state from positive to negative depending on the pH of the solution, as observed in Figure 1d, which depicts the apparent zeta-potential variation. Zein predominantly consists of α-zein, which has an isoelectric point at about 6.8; hence, at a pH below this value, zein is positively charged. At the same time, both polysaccharides were negatively charged irrespective of the tested pH value (Figure S4). Since our aim was to obtain interpolymer complexes, and taking into account the corresponding characteristics of the polymers in the solution, the pH of 4 was chosen, where the zein in 60 (Z60) and 70% (Z70) alcohol solutions was positively charged, complementary to the negative charges of both utilized polysaccharides. Therefore, these solutions were chosen for further use, with the attempt to obtain NPEC nanoparticles with the anionic polysaccharides.

Further considering the zein solution state, the DLS experiments (Figure S1) showed the presence of a single population of zein aggregates for mixtures with a 60 and 70% alcohol content, where several populations of aggregates can be observed in the rest alcohol–water mixtures. The results indicate that rather well-defined aggregates of zein exist at the particular solvent ratios chosen (the existence of zein aggregates is corroborated by the fact that the dimensions observed are much higher than the ones expected for single zein molecules). Further insights regarding the variation in the size of the individual aggregate populations with the pH are given by the results shown in Figure S2.

The 70% mixture (Z70) showed the smallest variation in the size with the pH. These mixed-solvent conditions for the zein were selected for further complexation studies with the CSA and ALG. Therefore, it should be kept in mind that the complexes prepared in the next step are formed between the cationic zein aggregates and the anionic polysaccharides, a feature that is expected to have some influence on the structure and properties of the final complexes/nanoparticles.

### 3.2. Characterization of the NPECs

**Figure 2:**
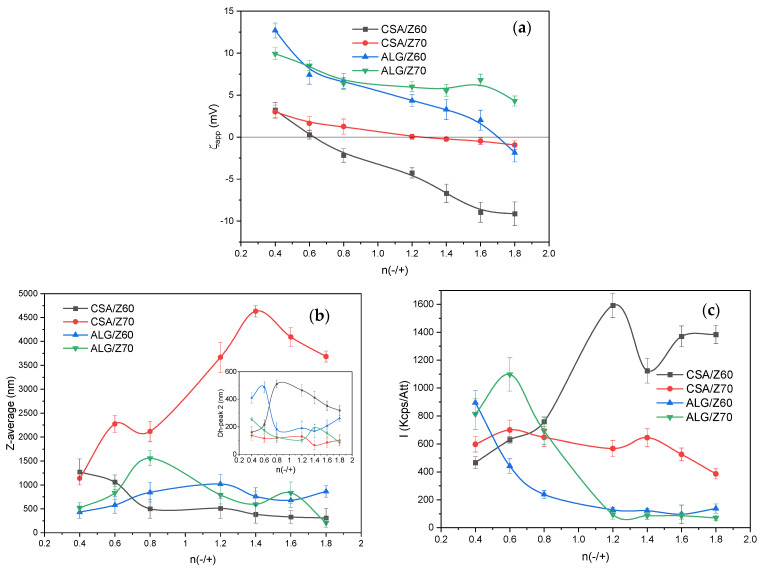
(**a**) The apparent zeta potential (ζ_app_), (**b**) Z-average particle size (inset—the mean diameter) and (**c**) the scattered light intensity of the polysaccharide/zein NPECs (addition rate = 0.08 mL·min^−1^). Error bars were calculated based on three independent measurements.

The influence of the polymer structure, molar mass, concentration and the titrant addition rate on the complex nanoparticle size, polydispersity and storage colloidal stability of the NPECs as colloidal dispersions have already been studied [36,37], mainly for synthetic polyelectrolytes. The titrant addition rate and the addition order of the components could define the particle sizes and polydispersity, as well as their electrokinetic behavior, and these factors can be used as a tool to tailor complex nanoparticles. However, the influence of these factors on the characteristics of the complex dispersions formed by natural polymers is rarely addressed in the literature. These insufficiently explored aspects prompted us to develop a systematic study on the influence of the polymeric component addition order, at two different addition rates, on the formation of the NPECs as colloidal dispersions based on the zein alcohol/water solutions (Z60 and Z70) and two anionic polysaccharides (CSA and ALG). The variation in the characteristics (the size, polydispersity and zeta potential) for the prepared NPECs when the titrate was zein were followed as a function of the polyanion type, the alcohol content in the zein solution and the charge molar ratio between the biopolymers utilized (Figure 2 and Figure S5).

The results presented in Figure 2a for the apparent zeta potential (ζ_app_) are atypical for NPEC systems, with the isoelectric point (n_iso_, the point of zero charges characteristic for stoichiometric PECs) not being found for the ALG/Z70 pair in the investigated molar ratio range (0.4–1.8). Theoretically, n_iso_ should be located at n(−/+) = 1; below and above this, the NPEC charges are provided by the polymer in excess. Nevertheless, the CSA-based NPECs show that n_iso_ is shifted to lower (n(−/+) = 0.6, CSA/Z60) or higher values (n(−/+) = 1.2, CSA/Z70), being influenced by the ionization degree of the zein and its ability to interact with the anionic polysaccharide. This behavior suggests that Z70, even with a lower ionization degree as compared to Z60, interacts with the CSA and forms almost-neutral nanoparticles (+/−2.5 mV) on the whole range of the investigated charge molar ratios, with a low dispersion stability. On the contrary, the same polysaccharide (CSA) interacts quickly and neutralizes the Z60 ionic charges much below the theoretical isoelectric point (at about n(−/+) = 0.6); after that, the CSA chains are found in excess. For the analogous Z60 titration with the ALG, the zeta-potential curve is similar in shape with that of the CSA, but shifted to more positive values, with an isoelectric point located way above the theoretical value (at about n(−/+) = 1.6). In this case, most probably the compensation of complementary ionic charges does not take place at low molar ratios; therefore, irregular structures are formed. The observed behavior may also be a consequence of the aggregation state of the zein at different solution conditions, taking into account that the aggregates may be prone to structural changes upon complexation.

All the above observations were sustained by the size and scattered light intensity values (Figure 2b,c), with the highest size and intensity being found for the CSA/Z70 series and the lower ionization degree (lower zeta-potential values) indicating a higher agglomeration degree, close to the upper measurement limit of the device. It was also observed that the particles obtained with the CSA and Z60 zein solution were smaller than those obtained with the Z70 solution. Also, the particle sizes depended on the ratio between the polymers and increased with the CSA content. A similar behavior was found for the ALG-based nanoparticles, with the particles being larger with the Z70 than with the Z60 at the same polymer ratio.

**Figure 3:**
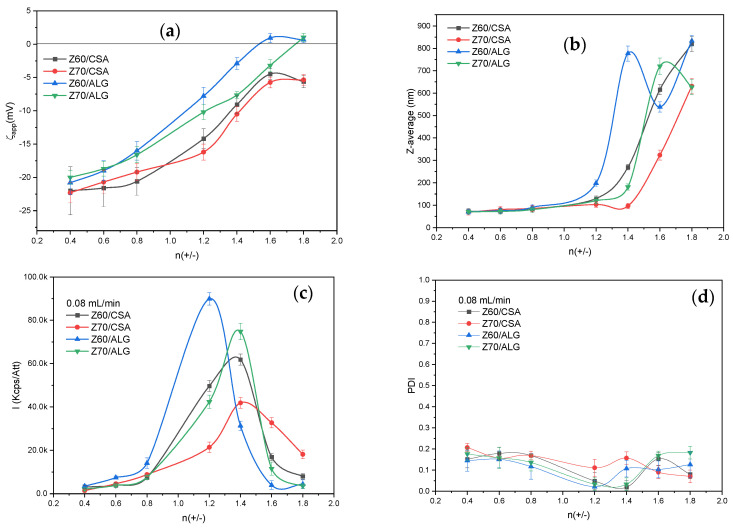
(**a**) The apparent zeta potential (ζ_app_), (**b**) Z-average particle size, (**c**) scattered light intensity and (**d**) polydispersity of the zein/polysaccharide NPECs (addition rate = 0.08 mL·min^−1^). Error bars were calculated based on three independent measurements.

The above-presented results may suggest that zein, at a very low concentration (10^−5^ M ionic units), may present elongated structures due to the low interchain interactions, which enable the intrachain electrostatic repulsions between similar ionic charges (positive in Z60 and Z70). The addition of the polysaccharides in the ten times higher ionic concentration (10^−4^ M ionic units) most probably leads to the formation of particles of a large size and with a broad size distribution (Figure 2a and Figure S5), even at a low charge ratio (n(−/+) = 0.4). In our attempt to decrease the particle size, the reverse addition order was tested, with the titrate being the polysaccharide and the titrant being the zein (Figure 3 and Figure S6).

As Figure 3a and Figure S6 clearly show, when the zein was the titrant, the particle size decreased drastically at molar ratios below 1.2, with a mean size of about 100 nm; also, almost monodisperse particles were obtained (Figure 3c, PDI below 0.2) compared with the particles prepared when the zein was the titrate (Figure 2a). The polysaccharide titrate can most probably engulf the more concentrated zein. Figure 3b shows a high increase in the scattering intensity when the charge molar ratio increased above 0.8, with an increase in the particle sizes too. As the size/intensity curves in Figure S6 show the formation of single populations with higher sizes and an even lower PDI, we may assume that, in this range, particle agglomerations in larger and denser structures are formed. This behavior is evident irrespective of the alcohol/water ratio and the polysaccharide structure. It may be assumed that the polysaccharide chain rapidly entrapped the zein dispersion and, even if the stoichiometry was not reached, the zeta-potential values were related to the available anionic groups on the surface of the formed particles (Figure 3d). Further decreasing the zein addition rate to 0.03 mL/min (Figures S7 and S8) did not impart drastic changes to the particle size and PDI, with scattered intensity values remaining at low values when the Z70 was used, irrespective of the polysaccharide utilized. This observation, along with the more negative values of ζ_app_, may suggest that the lower addition rate further enables the zein entrapment in the polysaccharide network.

**Figure 4:**
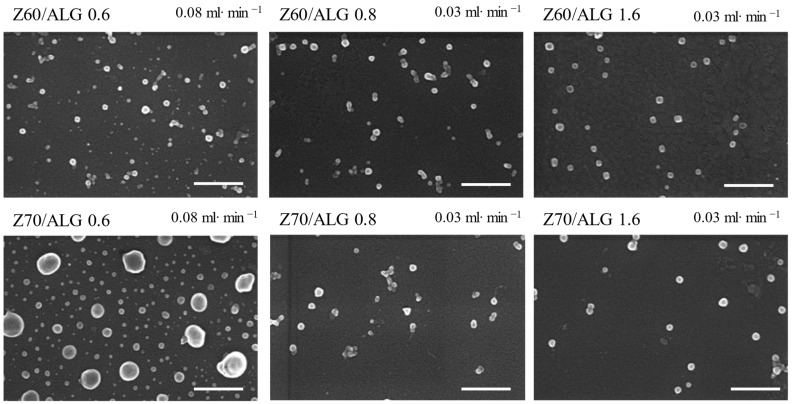
SEM micrographs of the selected ALG-based nanoparticles with different charge ratios between the biopolymers, different addition rates and different alcohol/water contents in the zein solutions (scale bar 500 nm).

**Figure 5:**
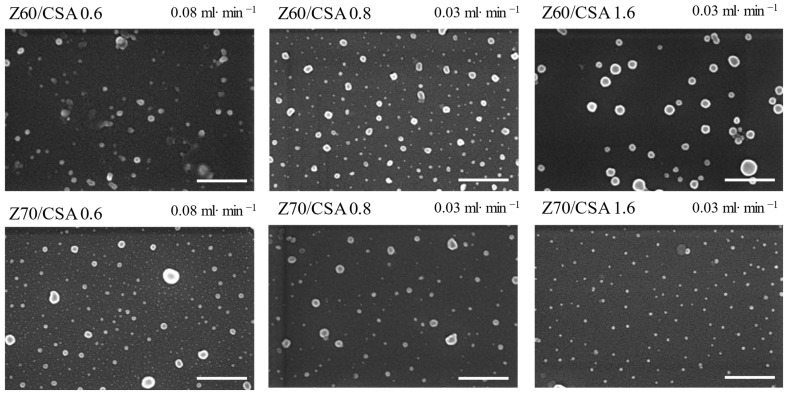
SEM micrographs of the selected CSA-based nanoparticles with different ratios between the biopolymers, different addition rates and different alcohol/water contents in the zein solutions (scale bar 500 nm).

The size and shape of the obtained nanoparticles when the zein was the titrant at both addition rates were characterized by SEM (Figure 4 and Figure 5).

Thus, the dispersed nanoparticles dried on the silicon wafer surface show mostly spherical shapes (mainly for the CSA-based samples), along with some irregularly shaped or agglomerated structures with sizes slightly smaller than those determined by the DLS measurements, probably due to the water loss during drying. Also, the color contrast at the particle edges could suggest a core/shell structure formation, supporting the assumption, based on the zeta-potential values, that the polysaccharide chains are engulfing the zein aggregates (Figure 3 and Figure S7).

**Figure 6:**
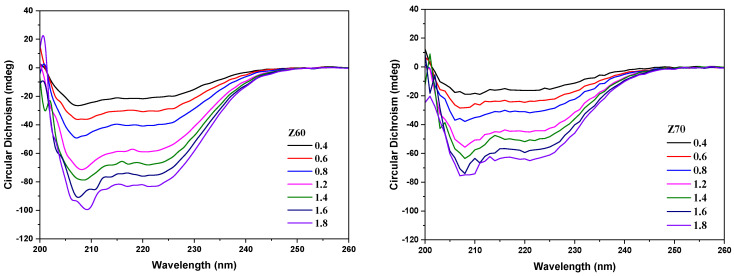
Circular dichroism spectra of the zein solutions with different protein concentrations (0.4–1.8) and different alcohol/water ratios (Z60 and Z70).

**Figure 7:**
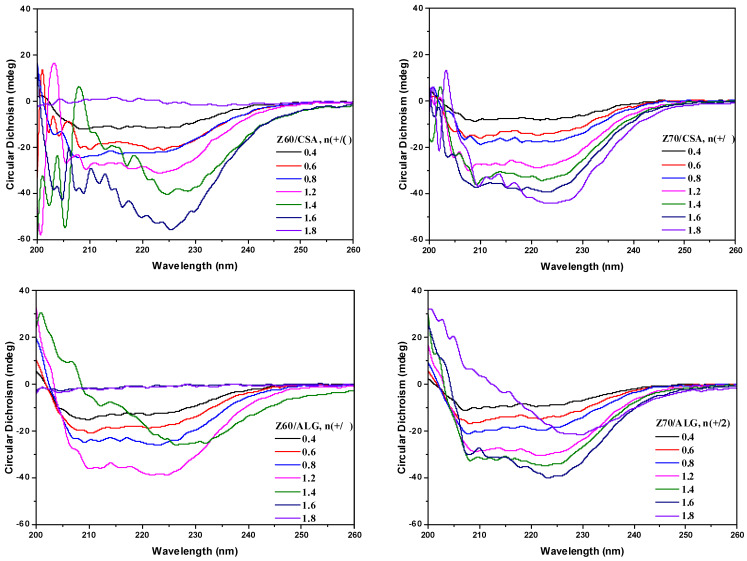
Circular dichroism spectra of the NPEC dispersions with different charge molar ratios, n(+/−) = 0.4–1.8, for both polyanions (ALG and CSA) and different alcohol/water ratios (Z60 and Z70).

The changes in the secondary structure of the zein macromolecules in the solutions (i.e., Z60 and Z70) and in the NPEC nanoparticles during complexation are also of scientific and practical interest, and have been investigated using circular dichroism (CD) spectroscopy (Figure 6 and Figure 7).

The CD spectra of the zein in the solution were measured under similar conditions as the amount added in the NPEC preparation, with the titrate solution being the corresponding alcohol/water solution (instead of the polysaccharide solution). The CD spectra of the zein show two negative peaks at ~208 and ~220 nm (Figure 6), which confirm the α-helix conformation of the zein molecules according to the literature [37], irrespective of the zein concentration or alcohol ratio. Also, the CD signal values of the corresponding peaks increased with the increase in the zein concentration and decreased with the alcohol/water ratio.

**Figure 8:**
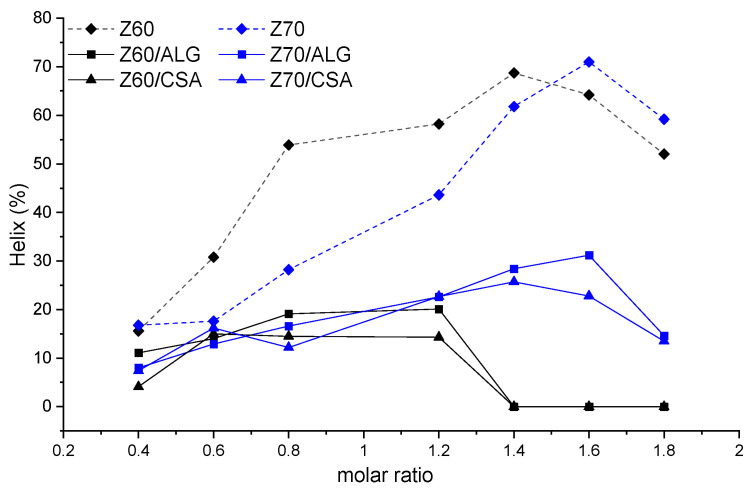
The content of α-helix in the zein solutions and the corresponding NPECs prepared with the CSA and ALG, taking into account the curves included in Figure 6 and Figure 7.

The change in the α-helix content of the zein molecules as a function of its content in polysaccharide-based NPECs is shown in Figure 7, which was qualitatively estimated from the intensities of the two negative peaks of the CD spectra. Surprisingly, the increase in the zein content (increasing the molar ratio between complementary biopolymers) resulted in the decrease in the intensities of the two negative peaks of the CD spectra, or even their disappearance, suggesting the decrease in the α-helix content. The addition of the zein in the CSA or ALG resulted in the reduced magnitudes of the corresponding peaks, indicating that the binding of the polysaccharide molecules may destroy the hydrogen bonds between the polypeptide chains of the zein molecules, leading to the decrease in the fraction of the helical structure of the zein protein. To further prove this assumption, the α-helix content in each sample was calculated and is illustrated in Figure 8.

Thus, the content of α-helix in the neat zein solutions (without polysaccharides) increased with the zein concentration. When the titrate was the polysaccharide solution, in the range of molar ratios n(+/−) = 0.4–1.2, the content of α-helix slightly varied in the range of 10–20%, irrespective of the zein solution used (Z60 or Z70). At higher molar ratios, the alcohol content in the zein solution strongly influenced the content of the α-helix structures; in the Z60 solutions, this led to the complete absence of secondary structures in the zein. In contrast, in the Z70 solutions, the zein continued to maintain its secondary structure of α-helix in the whole range of the investigated molar ratios. Increasing the alcohol content from 60 to 70% led to the decrease in the zeta potential (Figure 1) and, consequently, to a lower number of ionic sites available for electrostatic interactions with polysaccharide anionic charges; thus, the zein secondary structure was better preserved.

### 3.3. NPEC/Ciprofloxacin Composite Particles

Ciprofloxacin (CF) is a fluoroquinolone antibiotic with a spectrum activity on bacterial infections that includes intra-abdominal infections, respiratory tract infections and urinary tract infections, among others. Its combination with NPECs formed by natural macromolecules, such as those used in this study, is expected to result in materials with antibacterial activity, with applications which involve mainly surface coatings and antibacterial protection.

**Figure 9:**
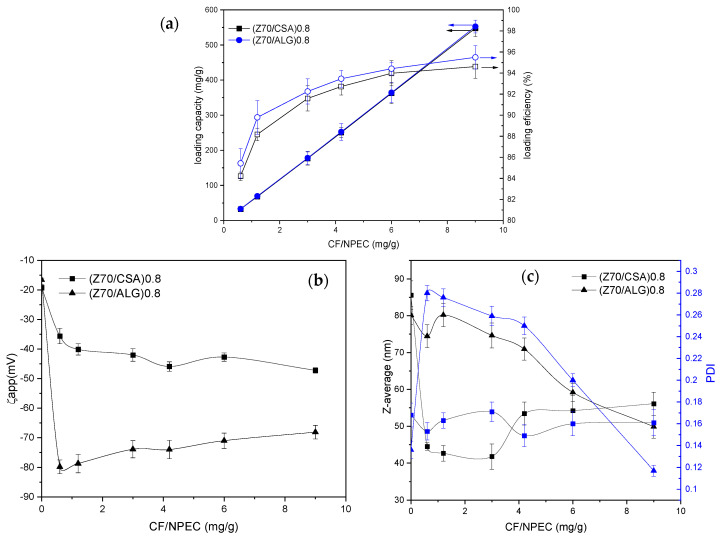
(**a**) The NPEC loading capacity (full symbols) and efficiency (open symbols), and (**b**) the apparent zeta potential and (**c**) particle size (Z-average), and the size dispersity, of the CF/NPECs as a function of the CF content.

**Figure 10:**
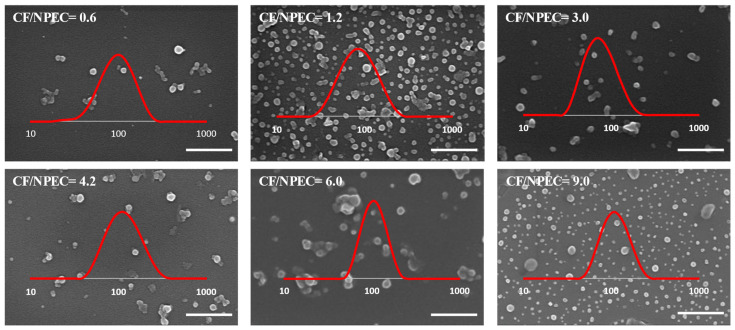
SEM micrographs of the CSA-based NPECS with n(+/−) = 0.8 and different CF contents, as shown in the images (scale bar 500 nm). Insets—particle size distribution determined by DLS (x-axis scale in nm).

Taking into account the previous results, two NPEC samples having similar zeta-potential values (about −20 mV, Figure 3) were selected, with n(+/−) = 0.8 and an addition rate of 0.03 mL· min^−1^, as well as Z70 and the two polysaccharides to be tested, with respect to the interaction with the CF drug. At the same time, at a working pH = 4, the CF was positively charged (Figure S9); therefore, we can assume that the electrostatic interactions are the predominant ones, but the hydrogen bonds and hydrophobic interactions with the zein and/or polysaccharide chains can occur. The loading capacity of the NPECs, as well as their loading efficiency, along with the characteristics of the NPEC/CF particles, are presented as a function of the CF content in Figure 9, whereas their morphology is shown in Figure 10.

As found in Figure 9a, the NPEC loading capacity increases with the initial concentration of the drug in the solution for the whole concentration region investigated, irrespective of the polysaccharide used in the NPEC preparation. Simultaneously, the loading efficiency increased from about 84 to 95 mg/g in the same CF concentration range, reaching about a 500 mg loading for the largest CF/NPEC ratio used. The zeta potential for the CF/NPECs based on the CSA decreased to higher negative values (around −40 mV) as compared to that obtained for the corresponding nanoparticles without the CF (around −20 mV), irrespective of the CF content. On the other hand, for the ALG-based NPECs, the presence of the CF led to an increase in the zeta-potential values up to −70 mV, with the absolute values slightly decreasing with the increase in the CF. For the NPECs with the ALG, increasing the CF content led to a decrease in the nanoparticle size, together with a decrease in the polydispersity, suggesting that the CF improves the nanoparticles’ physical properties by reducing the aggregation tendency. In contrast, in the case of the NPECs with the CSA, the size of the nanoparticles increased slightly with the increase in the CF content, resulting in CF/NPECs of a maximum of 60 nm. Even more, the polydispersity values of the NPECs with the CSA were below 0.2 for all samples regardless of the CF content, indicating almost monodisperse particles. The SEM micrographs in Figure 10 show that the morphology of the nanoparticles did not change after the interaction with the CF, suggesting a good incorporation of the antibiotic in the NPECs.

### 3.4. Antibacterial Properties of the CF/NPEC Particles

**Figure 11:**
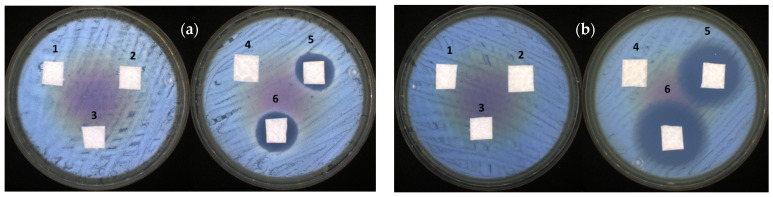
Antibacterial activity of the tested samples against (**a**) *S. aureus* and (**b**) *E. coli* of a superhydrophilic nonwoven material treated with Z70, NPECs or NPEC/CF (see the correspondence of the sample number in Table 2).

Aiming at exploring their biotechnological application, potential studies on the antibacterial properties of the CF/NPEC composite particles were undertaken, focusing on skin treatment approaches. The obtained dispersions were transferred onto the surface of a superhydrophilic nonwoven material (NWM) and tested against S. aureus and E. coli bacterial strains. The obtained results of the antibacterial properties for the bare NWM and that with Z70, NPECs or NPEC/CF are presented in Figure 11 and Table 2.

As can be seen, the pristine NWM, as well as the samples containing NPECs, had no antibacterial activity due to their excellent biocompatibility properties [38]. Only the samples with the added antibiotic presented antibacterial activity (up to 27 mm in the inhibition zone in the case of the NPECs with the CSA and ciprofloxacin against *E. coli*). It is known that ciprofloxacin is highly efficient against Gram-negative bacteria and moderately efficient against Gram-positive bacteria [39]. This is in accordance with the present antibacterial test, with the CF/NPECs having a larger inhibition area for *E. coli* than *S. aureus* in the composite particles, regardless of the polysaccharides involved (CSA or ALG), in the range of, or even larger than, other studies found in the literature (Table S1).

## 4. Conclusions

The preparation and characterization of nonstoichiometric polyelectrolyte complexes containing zein and two types of polysaccharides, namely, chondroitin sulfate A and sodium alginate, were presented. First, the behavior of the zein in different alcohol–water concentrations and at different pHs was evaluated, elucidating the aggregation state of the zein at different alcohol–water solutions. The NPECs were synthesized by two complementary addition methods and different addition rates, with a molar ratio between complementary charges ranging from 0.4 to 1.8. When the zein was the titrant, and at molar ratios below 1.2, particles with a mean size of about 100 nm were obtained, being almost monodisperse (a PDI below 0.2). When the zein was the titrate, higher sizes and size polydispersity were obtained. At the same time, the polysaccharide titrate could most probably easily engulf the more concentrated zein, forming core–shell structures. The decrease in the α-helix content of the zein as a function of its content in the NPECs was observed, with the binding of polysaccharide chains most probably destroying the hydrogen bonding between the polypeptide chains of the zein protein, leading to the decrease in the fraction of the protein helical structure. The NPECs with the best characteristics (a 0.8 molar ratio between polymers, 100 nm in size, −20 mV zeta potential) were successfully used as nanocontainers of ciprofloxacin. The CF/NPEC antibacterial activity was evaluated against *S. aureus* and *E. coli* by treating a nonwoven cellulose material with the biomacromolecule/drug complex solution. The CF/NPECs showed a high antibacterial activity against both bacterial strains, being more pronounced in the case of Gram-negative *E. coli*, regardless of the polysaccharide utilized.

## Figures and Tables

**Table 1:** Sample codes and preparation conditions.

Sample Code	Titrant, 10^−4^ M (mL)	Titrate, 10^−5^ M (mL)	Charge Molar Ratio, n(+/−) or n(−/+)	Addition Rate, mL·min^−1^
(Zxx/PZ)0.4 or (PZ/Zxx)0.4	1.2	30	0.4	0.03/0.08
(Zxx/PZ)0.6 or (PZ/Zxx)0.6	1.8		0.6	
(Zxx/PZ)0.8 or (PZ/Zxx)0.8	2.4		0.8	
(Zxx/PZ)1.2 or (PZ/Zxx)1.2	3.6		1.2	
(Zxx/PZ)1.4 or (PZ/Zxx)1.4	4.2		1.4	
(Zxx/PZ)1.6 or (PZ/Zxx)1.6	4.8		1.6	
(Zxx/PZ)1.8 or (PZ/Zxx)1.8	5.4		1.8	

xx is the alcohol concentration in the zein (Z) solution (60 or 70%); PZ is the ALG or CSA.

**Table 2:** Antibacterial activity of the tested samples against the reference strains.

Sample No.	Sample Code	Inhibition Zone (mm)	
		*S. aureus*	*E. coli*
1	NWM	-	-
2	NWM + Z70 10^−4^ M	-	-
3	NWM + (Z70/ALG)0.8	-	-
4	NWM + (Z70/CSA)0.8	-	-
5	NWM + CF/(Z70/ALG)0.8 = 9	21.35 ± 2.33	25.15 ± 1.20
6	NWM + CF/(Z70/CSA)0.8 = 9	20.10 ± 0.57	27.75 ± 1.20

## Data Availability

The data presented in this study are available on request from the corresponding authors.

## Supplementary Materials

The following supporting information can be downloaded at: https://www.mdpi.com/article/10.3390/nano14020197/s1, Figure S1: Size distributions by the DLS of the zein protein at different alcohol–water mixtures and at different pH values; Figure S2: Variation in the aggregate size in the zein solutions at different alcohol–water mixing ratios and pH values from the DLS experiments; Figure S3: Acidic and basic amino acids in the zein; Figure S4: ζ_app_ vs. pH for the two polysaccharides utilized in this study, sodium alginate (ALG) and chondroitin sulfate (CSA); Figure S5: Size distributions from the DLS for the zein/polysaccharide complexes at different solvent mixtures and at different charge ratios, n(−/+); Figure S6: Size distributions from the DLS for the zein/polysaccharide complexes at different solvent mixtures and at different charge ratios, n(−/+); Figure S7: Size distributions from the DLS for the zein/polysaccharide complexes at different solvent mixtures and at different charge ratios, n(−/+); Figure S8: Variation in the size (D_h_-average), light-scattering intensity (I), size polydispersity index (PDI) and zeta potential (ζapp) with n(−/+) for different zein/PZ complexes formed under different solvent and mixing conditions with the addition rate of 0.03 mL/min; Figure S9: Potentiometric titration of ciprofloxacine vs. pH. Table S1: Literature data as compared with the results obtained in this work considering the materials performances as concern the inhibition zone against the reference strains.
