# Supplementary material for: Zein/Polysaccharide Nanoscale Electrostatic Complexes: Preparation, Drug Encapsulation and Antibacterial Properties
Source: Nanomaterials (Basel). 2024 Jan 15;14(2):197. doi: 10.3390/nano14020197 (PMC10818850; doi:10.3390/nano14020197)
Supplement: Supplementary file 1 [file nanomaterials-14-00197-s001.zip › nanomaterials-2771252-supplementary.pdf]

nanomaterials

MDPI

Supplementary Materials

Zein/Polysaccharide Nanoscale Complexes: Preparation, Drug Encapsulation and Antibacterial Properties

Elena-Daniela Lotos,[1] Marcela Mihai,[1,*] Ana-Lavinia Vasiliu,[1] Irina Rosca,[1] Alice Mija,[2] Bogdan C. Simionescu,[1] Stergios Pispas[1,3,*]

[1]Petru Poni Institute of Macromolecular Chemistry, 41A Grigore Ghica Voda Alley, 700487, Iasi, Romania
[2]Université Côte d'Azur, Institut de Chimie de Nice, UMR CNRS 7272, 28 Av Valrose, Nice, France
[3]Theoretical and Physical Chemistry Institute, National Hellenic Research Foundation, 48 Vassileos Constantinou Ave, 11635 Athens, Greece

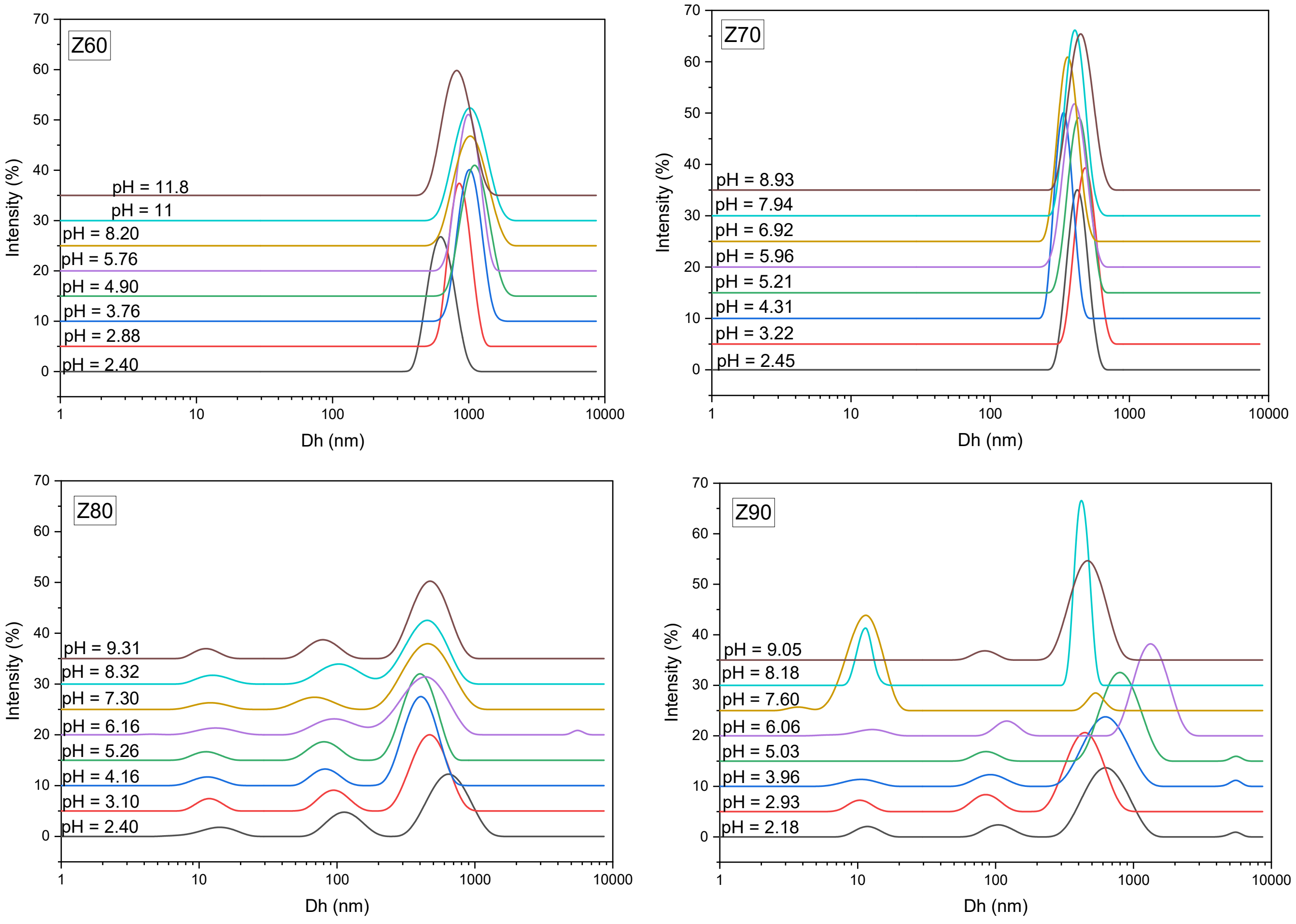

Figure 1S. Size distributions by DLS of zein protein at different alcohol-water mixtures and different pH values. Zxx in the upper left inset denotes the alcohol % content of the solvent mixture.

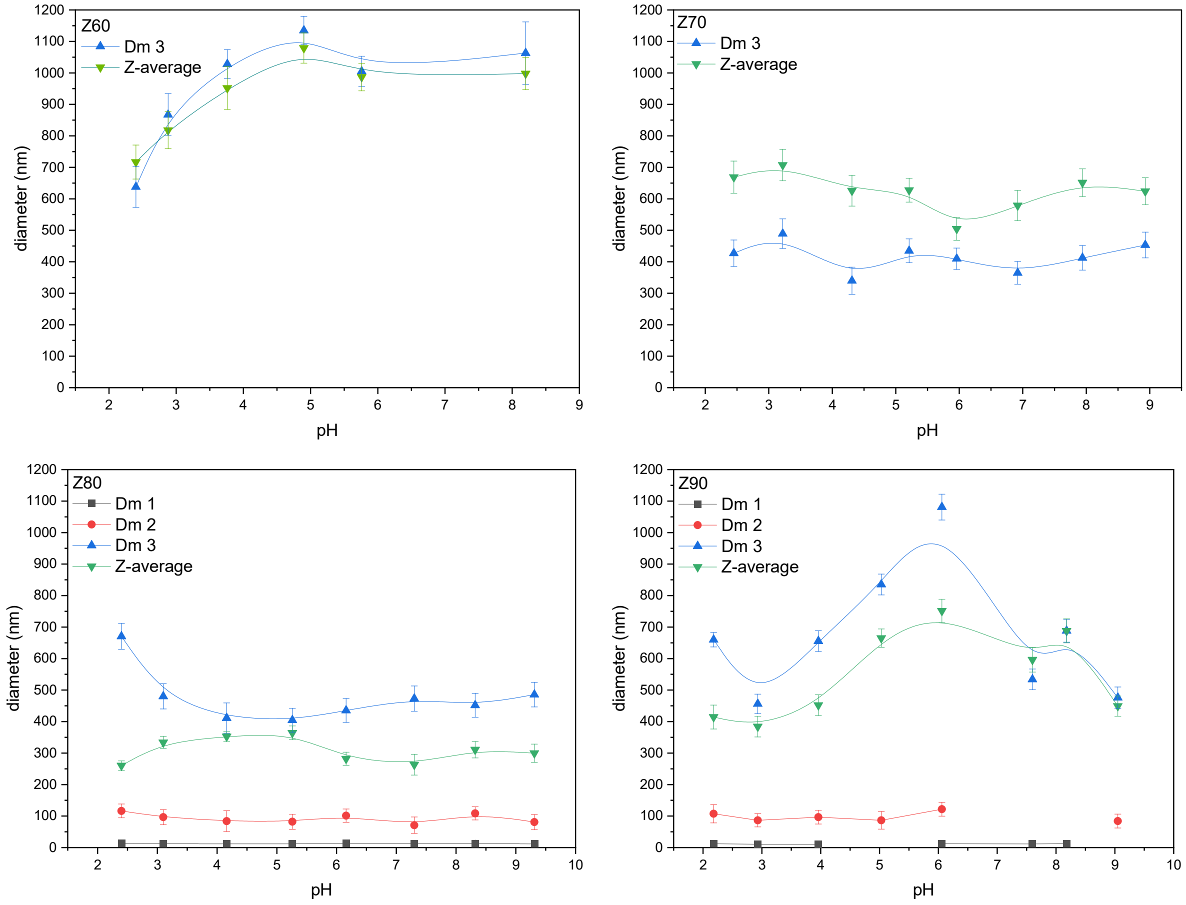

Figure 2S. Variation of aggregate size in zein solutions at different alcohol-water mixing ratios and pH values from DLS experiments. Z-average is the peak position size of the aggregates from cumulant analysis. Dm is the diameter of each aggregate population observed in each sample. Dm 3 is the mean diameter of the peak ascribed to the largest size aggregates for each sample. Error bars were calculated based on three independent measurements.

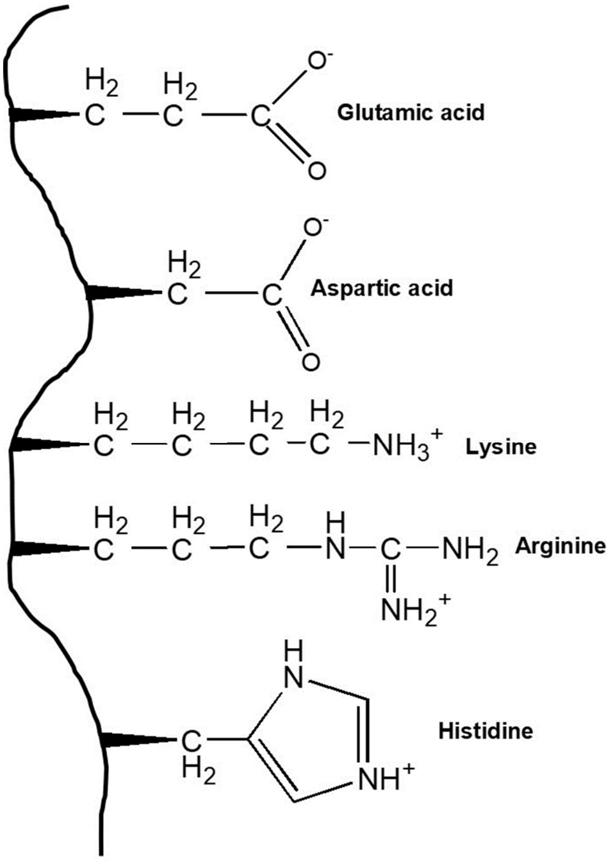

Figure 3S. Acidic and basic amino acids in zein.

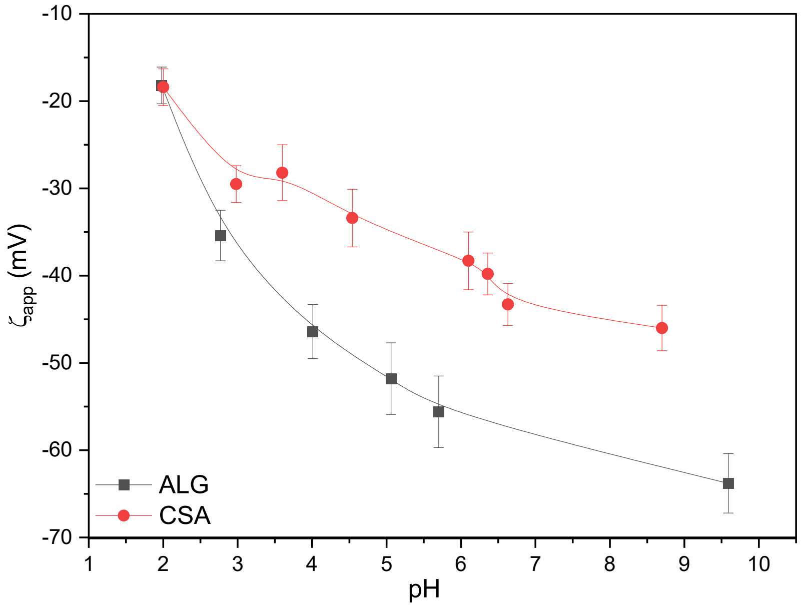

Figure 4S. ζ_{app} vs pH for the two polysaccharides utilized in this study, sodium alginate (ALG) and chondroitin sulfate (CSA). Error bars were calculated based on three independent measurements.

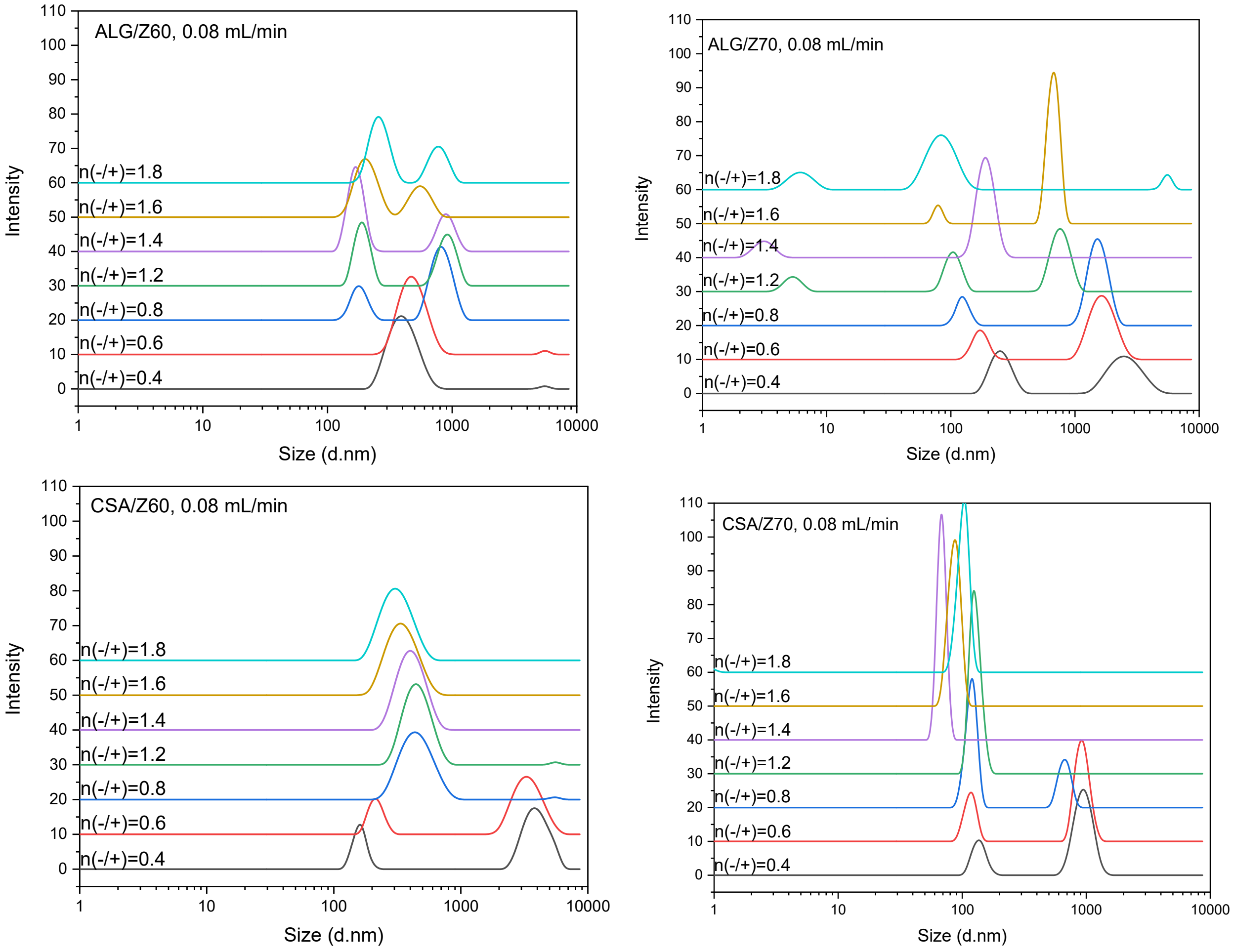

Figure 5S. Size distributions from DLS for zein/polysaccharide complexes at different solvent mixtures and different charge, n(-/+), ratios. Conditions: PZ solution added to zein solution, addition rate 0.08 mL/min. The presence of monomodal size distributions for zein/CSA complexes at higher n(-/+) is observed.

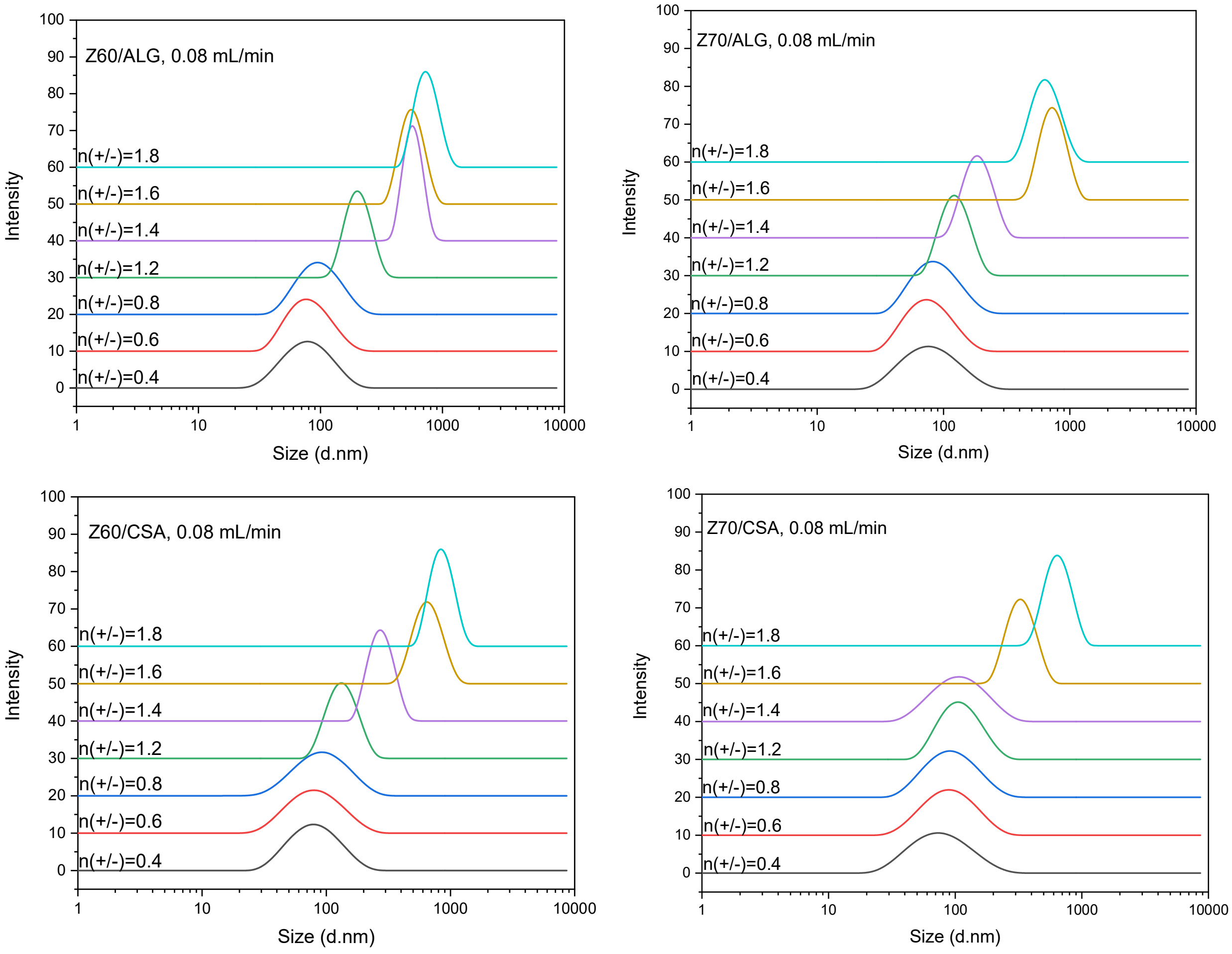

Figure 6S. Size distributions from DLS for zein/polysaccharide complexes at different solvent mixtures and different charge, n(-/+), ratios. Conditions: zein solution added to PZ solutions, addition rate 0.08 mL/min. The presence of monomodal size distributions for all zein/PZ complexes is notable. Larger sizes, close to micron range, are obtained at higher n(-/+) ratios.

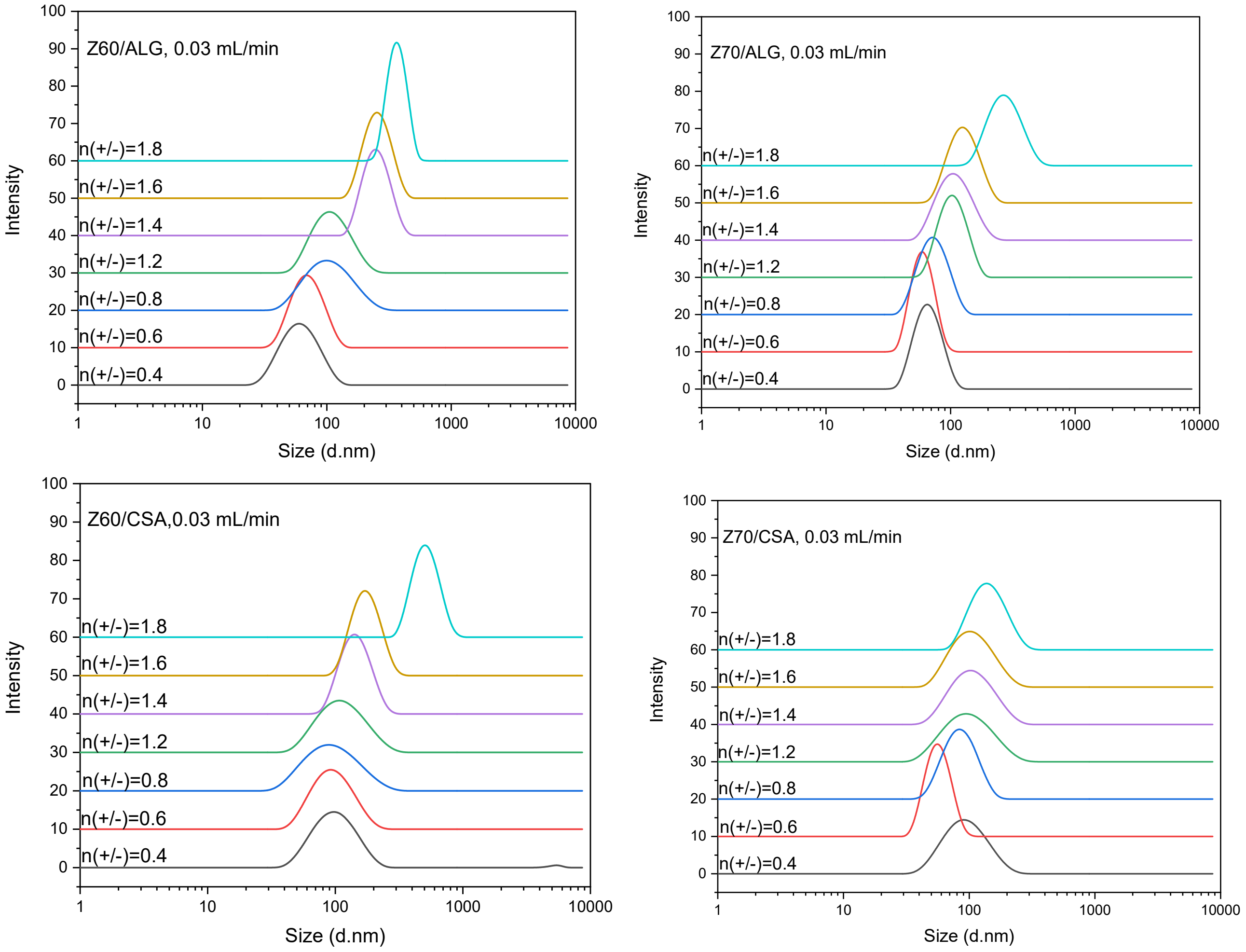

Figure 7S. Size distributions from DLS for zein/polysaccharide complexes at different solvent mixtures and different charge, n(-/+), ratios. Conditions: zein solution added to PZ solutions, addition rate 0.03 mL/min. The presence of monomodal size distributions for all zein/PZ complexes should be noted. Smaller sizes are obtained at this addition rate.

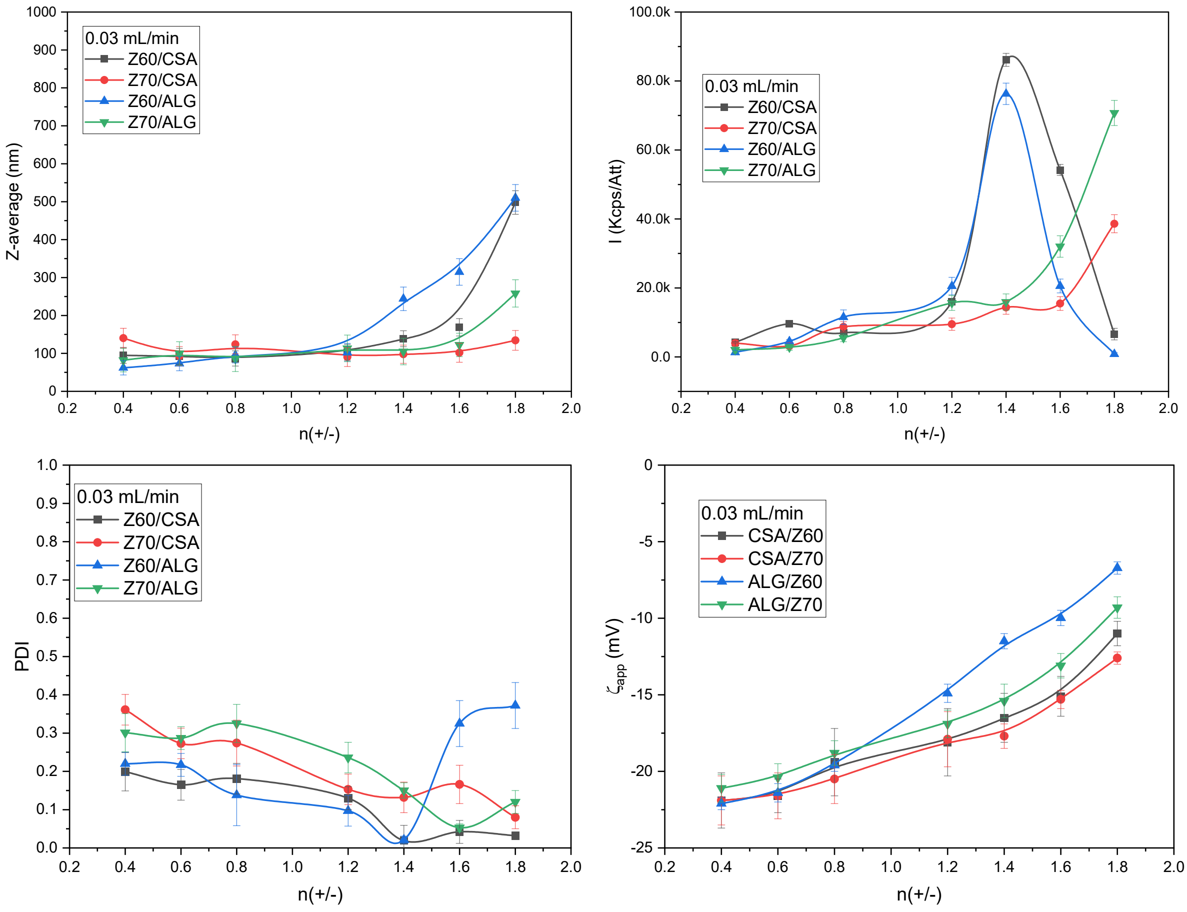

Figure 8S. Variation of size (Dh-average), light scattering intensity (I), size polydispersity index (PDI) and zeta potential (ζapp) with n(-/+) for different zein/PZ complexes formed under different solvent and mixing conditions, for the addition rate of 0.03 mL/min. Error bars were calculated based on three independent measurements.

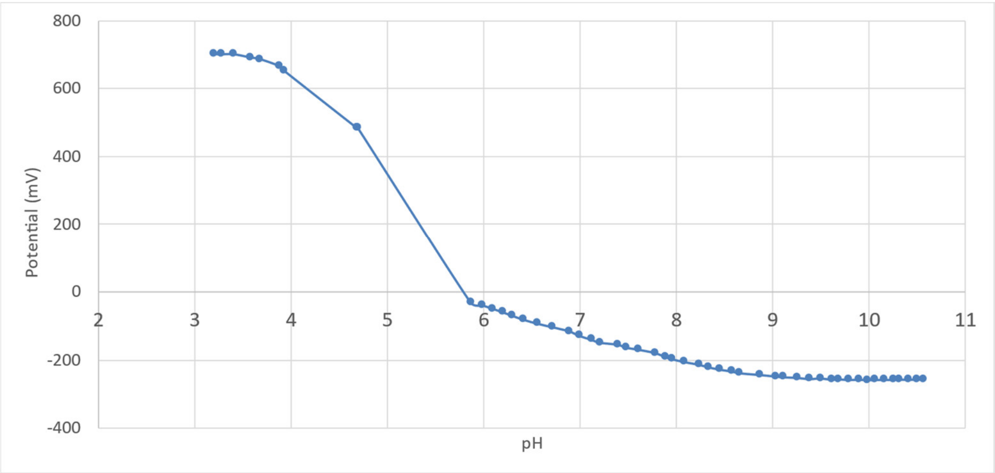

Figure 9S. Potentiometric titration of ciprofloxacine vs pH

Table 1S. Literature data as compared with the results obtained in this work considering the materials performances as concern the inhibition zone against the reference strains

**Inhibition zone**		**Reference**
**S. aureus**	**E. coli**	
20	19	https://doi.org/10.1002/jbm.b.31615
22	26	https://doi.org/10.3390/pathogens5010028
10	11	https://doi.org/10.1080/16878507.2020.1748941
12	18	https://doi.org/10.1080/14756360310001624948
21	22	https://doi.org/10.1021/acsmedchemlett.5b00146
9.3	19.8	https://doi.org/10.1007/s00284-012-0094-7
**21.3**	**25.1**	**NWM + CF/(Z70/ALG)0.8 =9 - this work**
**20.1**	**27.7**	**NWM + CF/(Z70/CSA)0.8 =9 - this work**
